# Patients’ perceptions and practices of informing relatives: a qualitative study within a randomised trial on healthcare-assisted risk disclosure

**DOI:** 10.1038/s41431-024-01544-8

**Published:** 2024-02-02

**Authors:** Charlotta Nääs, Jenny von Salomé, Anna Rosén

**Affiliations:** Department of Diagnostics and Intervention, Oncology, Umeå University, Umeå, Sweden

**Keywords:** Preventive medicine, Outcomes research, Genetic counselling, Genetic testing, Genetics research

## Abstract

In a multicentre randomised controlled trial (DIRECT), we evaluate whether an intervention of providing direct letters from healthcare professionals to at-risk relatives (ARRs) affects the proportion of ARRs contacting a cancer genetics clinic, compared with patient-mediated disclosure alone (control). With the aim to explore how the patients included in the trial perceived and performed risk communication with their ARRs we analysed 17 semi-structured interviews with reflexive thematic analysis. All patients described that they disclosed risk information to all close relatives themselves. No integrity-related issues were reported by patients offered the intervention, and all of them accepted direct letters to all their ARRs. Patients’ approaches to informing distant relatives were unpredictable and varied from contacting all distant ARRs, sharing the burden with the family, utilising the offer of sending direct letters, vaguely relying on others to inform, or postponing disclosure. Most patients limited their responsibility to the disclosure, although others wanted relatives to get genetic counselling or felt a need to provide additional information to the ARRs before ending their mission. We also identified confusion about the implication of test results, who needed risk information, and who was responsible for informing ARRs. These misunderstandings possibly also affected risk disclosure. This study revealed that despite accepting the direct letters to be sent to all relatives, the patients also contributed to risk disclosure in other ways. It was only in some situations to distant relatives that the healthcare-assisted letter was the only means of communication to the ARRs.

## Introduction

### Identifying cancer predisposition enables prevention

Targeted prevention programs for individuals with hereditary risk for breast cancer, ovarian cancer and colorectal cancer (CRC) are cost-effective and reduce both cancer incidence and mortality [1–3]. In Sweden, as in most European countries, cancer genetic clinics rely on family-mediated disclosure of hereditary risk—i.e., healthcare professionals (HCP) encourage patients with a pathogenic variant (PV) in a cancer-predisposing gene to inform their family members about the options of counselling, testing and surveillance. The majority of at-risk relatives (ARRs) are informed about their risk [4, 5], but communication barriers may result in active or passive non-disclosure [6]. A recent meta-analysis on the uptake of counselling in *BRCA1*, *BRCA2*, and Lynch families showed that when applying a family-mediated disclosure strategy, 35% of ARRs received genetic counselling, while direct contact from healthcare to ARRs increased the uptake to 63% [7]. The studies in the meta-analysis are heterogeneous and some performed in a research setting, and a recently published observational report found no evidence of increased uptake after implementing a proactive approach into clinical practice [8].

### Understanding patient perspectives when evaluating disclosure in a randomised trial

During 2020–2023, we recruited participants with a hereditary risk of breast cancer, ovarian cancer, or CRC to a pragmatic multicentre, randomised controlled trial at Swedish cancer genetics clinics. The aim of the RCT is to evaluate whether the additional offer of sending direct letters to eligible ARRs (intervention group) increases the proportion of ARRs contacting a cancer genetics clinic within 12 months, as compared to family-mediated risk disclosure alone (control group).

Risk disclosure between HCPs, the patients and their ARRs is a complex process of interactions and thus, also difficult to study. In this qualitative study within a trial, we intended to decipher part of this complex process by exploring how the patients involved in DIRECT perceived and practised risk disclosure to their ARRs.

## Material and methods

### Setting

In Sweden, hereditary cancer care is centralised in cancer genetics clinics at university hospitals. These clinics provide pre-and post-test genetic counselling of patients with familial aggregation of cancer, post-test counselling of patients with germline PV detected in mainstream testing and genetic counselling to relatives requesting predictive testing. Patients with a germline PV are offered surveillance according to national guidelines [9, 10]. Individuals in families with high occurrence of breast cancer or CRC, but negative genetic screening results, can also be offered surveillance for early detection (familial breast cancer/familial CRC) [9, 10].

### DIRECT—a pragmatic clinical trial

DIRECT was designed to evaluate an intervention (offer of sending a direct letter to ARRs) given within clinical praxis according to local routines. Consenting adults were included if they had one of the following diagnoses: familial breast cancer, familial CRC, or a PV in *BRCA1*, *BRCA2*, *PALB2*, *MHL1*, *MSH2, MSH6*, or *PMS2* and had at least one eligible ARR (i.e., a relative deemed to be recommended genetic counselling within a year according to the involved HCP).

At all study sites, the patients received post-test counselling with a genetic counsellor and/or clinical geneticist through digital/telephone meetings or in-person visits. During counselling, the patients received information about their cancer genetic family investigation, including test results and potential preventive options for both the patient and ARRs. At some study sites, standard care included providing the patient with a ´family letter’ summarising the information, whereas others did not offer this service.

Per current standards, patients in both study arms were encouraged to inform ARRs about their potential risk (i.e., family-mediated disclosure). In collaboration, the patient and HCP listed the ARRs and if available, their contact details. In addition, the patients in the intervention group were offered direct letters to be distributed by the HCP to their ARRs 1 month later. If the patient approved of the intervention, letters were sent to the ARR(s) who were eligible for predictive testing or surveillance within the upcoming year. The direct letters were sent with registered mail and included information on the cancer risk assessment conducted in the family and possible implications for the ARRs. Further details of the study, including templates of the letters, are found in the published study protocol [11].

### Data collection and qualitative analysis

Qualitative data, collected in 2021 and 2022 through semi-structured telephone interviews were analysed using reflexive thematic analysis [12, 13]. The analysis resulted in the identification of subthemes and themes reflecting how the patients perceived and performed risk disclosure. Although this enquiry was rooted in qualitative data, we compared the data from each interview with RCT- data and categorised each patient’s risk disclosure approach(es) to close and distant relatives. In the text, we use the term ‘few’ when 1–2 patients expressed a certain thought or feeling, ‘some’ for 3–7, ‘many’ for 8–16, and ‘all’ if it was seen among all patients. The supplementary information provides details on the patient recruitment process, data collection, analytical procedure, and interview guides.

## Results

**Fig. 1:**
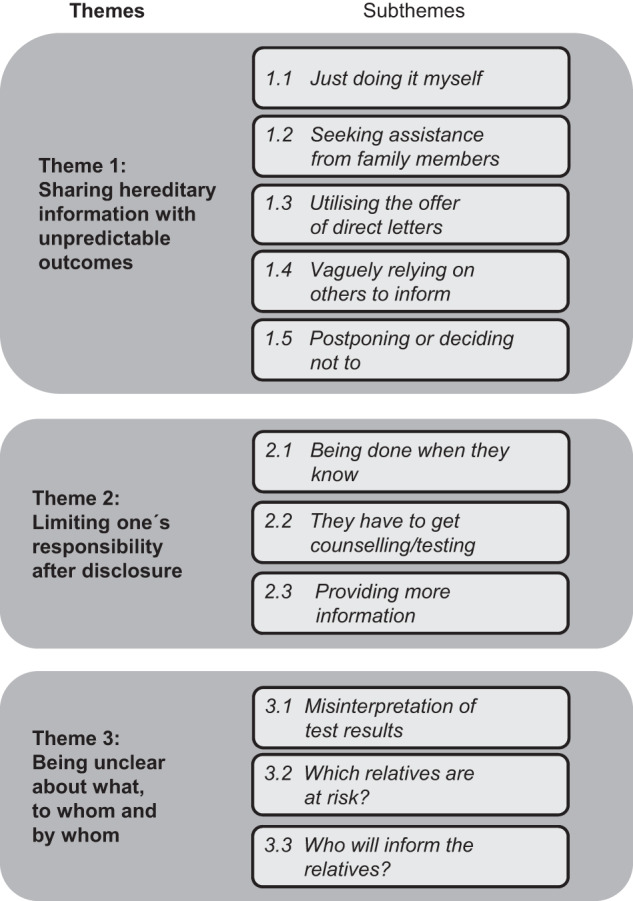
Overview of results. Themes and subthemes reflecting how the patients perceived and performed risk disclosure to at-risk relatives (ARRs).

Characteristics of the 17 patients (10 allocated to intervention and 7 to control group) are found in Table 1. The reflexive thematic analysis resulted in three themes described below (Fig. 1) reflecting how the patients’ perceived and performed risk disclosure to ARRs. The theme “**Sharing hereditary information with unpredictable outcomes**” consists of the patients’ motivation to share information, but dependent on their personal preferences and the intended audience the outcome of risk disclosure was unpredictable. The theme “**Limiting one´s responsibility after disclosure**” reflects that while most patients considered to ‘be done’ when the ARRs were informed, others extended their responsibility to be further engaged in the care of the ARR. Misunderstandings are conceptualised in the theme “**Being unclear about what, to whom and by whom**”. The last section covers a description of the patients’ experiences of participating in DIRECT. Quotations associated with the themes and subthemes are presented in Table 2.

**Table 1:** Characteristics of the participants.

	Subgroup	No
Gender	Female	10
	Male	7
Age	18–29	1
	30–39	2
	40–49	2
	50–59	5
	60–69	4
	70–75	3
Highest education	Highschool or less	3
	Up to 2 years of post-secondary level	8
	>2 years of post-secondary level	6
Cancer history	Yes	13
	No	4
Genetic testing	Screening	13
	Predictive testing	4
Family diagnosis	Familial breast cancer (Negative screening of breast cancer gene panel, but surveillance with yearly mammograms offered to ARRs)	6
	Familial colorectal cancer (Negative screening of colorectal cancer gene panel, but surveillance with colonoscopy every 5th year offered to ARRs)	3
	Lynch syndrome (Predictive testing of pathogenic variant in *MLH1, MSH2, MSH6* or *PMS2* offered to ARRs)	3
	Hereditary breast and ovarian cancer (Predictive testing of pathogenic variant in *BRCA1*, *BRCA2*, or *PALB2* offered to ARRs)	5
Study allocation	Control	7
	Intervention	10

**Table 2:** Themes and subthemes with corresponding quotations.

**THEME 1. Sharing hereditary information with unpredictable outcomes**
**1.1 Just doing it myself**
‘It was hard of course, to tell … that it’s like, that it’s genetic and things like that. But what to do, you must tell them, so there was nothing else to do.’ (# 6, female, intervention)
‘When I got this message that they had found a gene variant, uh, when I got it then, well I just called and talked to my sisters and told them how it was, so it was not a conversation that stands out, not like I remember they thought it was sad or upsetting or something like that, no it was very calm and matter-of-fact and so on.’ (# 15, male, intervention)
‘It felt like I did a great thing for everybody, so I took it that way. It was also well received; I didn’t feel uncomfortable but was just eager to share the information with everybody.’ (# 7, female, control)
I think maybe if I had not had cancer before and not gone down that bumpy road, I think maybe it could have been more difficult. But now I had started getting treatment, and I had to tell my family very tough things. Somehow it has opened up the doors for ongoing communication… it wasn’t that hard at that point of the whole cancer process, it felt like it was just another thing…” (# 11, female, intervention)
Interviewer (I): ‘How did you contact these 80-year-old cousins?’ Patient (P): ‘Well, by phone. One lives in [city] and one lives in [town].’ I: ‘Okay. Are you usually in contact with them?’ P: ‘Well, no I can’t say I am.’ I: ‘How did it feel to call them up and say something like this?’ P: ‘Well, that’s no problem. There are absolutely no worries. I have absolutely no problem with that.’ (# 18, male, control)
**1.2 Seeking assistance from family members**
‘I talked a lot with the aunt who had had it [cancer] and who wanted me to do the investigation, and we decided that I’d send the same information via email to everyone’. (# 2, female, control)
**1.3 Utilising the offer of direct letters**
‘I was asked if I would participate in this study when they sent out written information to the closest family and then I think I said that I will probably talk to the family anyway…but it is good that there is information both in writing and that I can tell them because I may have misunderstood something or forget to say something.’ (#11, female, intervention)
‘Well, I have talked to my siblings and my mom talked to the cousins, my cousins that is, but then the rest, whom I have basically never met, they [cancer genetics clinic] took care of sending to.’ (# 10, female, intervention)
‘I haven’t met them since we had family dinners with grandma when we were like under 15, so 40 years have passed and we’ve never even… well, I’d hardly recognised them, so it would probably feel a little strange to just call and tell them about something like this, then you’d probably rather have someone who knows what it’s about and can answer questions more easily. You also don’t know how they would react like if they would get worried or not because you don’t know them at all. So, it felt like when [my sister] said that ’well I think I don’t want to do it either’, then we were in complete agreement that, well, then you [cancer genetics clinic] can contact them, you can solve it.’ (#15, male, intervention)
**1.4 Vaguely relying on others to inform**
Interviewer (I): ‘You mentioned that you have relatives, was it cousins, that you have not had contact with for 40 years?’ Patient (P): ‘Yes, yes.’ I: ‘Have you considered ‘should we contact them’ or …?’ P: ‘Well, I didn’t think that was on me. I didn’t start this so I thought my sister can look into it if she feels she needs to.’ (#14, male, control)
Interviewer (I): ‘So, were those all the ones you know about, or are there some you haven’t talked to but are aware of, or how…?’ Patient (P): ‘Oh my, I have a lot of cousins… (laughs) A lot of cousins… (laughs) But these are the ones I’ve had the most contact with. Those who are a bit more scattered, I haven’t had much contact with, but they’ve probably received information from my cousins who are… Well, it was like a popcorn pot down in southern Sweden for a while.’ (#16, male, control)
**1.5 Postponing or deciding not to**
‘Well, I feel a little guilty because I should have sent … this letter [family letter provided by the cancer genetics clinic] […] it hasn´t been done and it is a pity because it should have been done, but I haven’t done it unfortunately.’ (# 4, female, control)
‘It might sound bitter and whatnot but those aunts, the others, they hurt me and my brothers so bad when my father passed away so I don’t feel I have any obligation even informing them […] No, I can’t even make myself want to contact them and tell them.’ (# 6, female, intervention)
**THEME 2. Limiting one’s responsibility after disclosure**
2.1 Being done when they know
‘I don’t know how many of those they sent letters to that took a test and found out, but at least I feel I have done what I can do, and that people have received information. […] Whether they want to find out or not, it’s up to them. […] That’s that, I can’t do more than that.’ (# 10, female, intervention)
‘Straight after getting tested and when they [the cancer genetic clinic] called, I phoned the boys right away [and said], ‘you should go get tested too’. I don’t know if all the boys have gotten tested.’ (# 13, male, intervention)
2.2 They have to get counselling/testing
‘It was a big step, but with a little motivation and help from his partner and our family he [the patient’s brother] did it [got tested].’ (# 8, female, intervention)
‘Well, as parents, we’ve been nagging them to do what they’re supposed to. Because it is in our interest that they get it done. Some of them have not been in too much of a hurry, but they have done it.’ (# 19, male, intervention)
2.3 Providing more information
‘So, we had another phone meeting a little further on, so they [the patient’s daughters] could ask about things they were thinking about, because I didn’t think I had the answers.’ (# 12, female, intervention)
**THEME 3. Being unclear about what, to whom and by whom**
3.1 Misinterpretation of test results
‘But I’ve told him I don’t carry the gene so that there are then, there are no worries.’ (# 17, male, intervention, with negative screening of colorectal cancer gene panel, but surveillance with colonoscopy offered every 5th year to ARRs)
3.2 Which relatives are at risk?
‘Well, you see, we’ve been working together to map out relatives with different last names but who are still related to us, through marriages and such. And it turns out that the other in-laws in our family, they have been free from this gene. It’s only our family on my dad’s side that has been affected, every other person it seems. And on my mom’s side, there hasn’t been anyone carrying this gene. Nope!’ (#19, male, intervention, overestimating the number of ARRs and thinking that also those married into the family are at risk)
3.3 Who will inform the relatives?
Patient (P): ‘And then they sent the paper, and we got power of attorney that we could check their medical records.’ Interviewer (I): ‘Right. And …’ P: ‘Well, I spoke to a cousin the other day and they hadn’t heard anything more, but …’ I: ‘How, you’re thinking something more about how the assessment went?’ Patient: ‘Well, yeah.’ (# 16, male, control, anticipating that the clinic would reach out to the relatives that sent in consent for checking their medical records to confirm cancer diagnoses among family members)

### Theme 1. Sharing hereditary information with unpredictable outcomes

In general, patients in both study arms described feeling responsible for making information available to ARRs, but the approach to risk disclosure depended on their own preferences and if the intended audience were close or distant relatives. The different strategies for risk disclosure (subthemes) resulted in both active and passive disclosure as well as non-disclosure. To allow for a comparison of the chosen approach by study arm and gender, the frequency of described disclosure strategies (subthemes) to close and distant relatives is presented in Table 3.

**Table 3:** Disclosure strategies when the patients approached close and distant relatives.

				Disclosure strategies^b^				
Study group	ID	Sex	ARR^a^	1.1 Just doing it myself	1.2 Seeking assistance from family members	1.3 Utilising the offer of direct letters	1.4 Vaguely relying on others to inform	1.5 Postponing or deciding not to
INTERVENTION	I #6	F	C	C (D)	-	C	(D)	(D)
	I #8	F	C	C	-	C	-	-
	I #9	F	C	C	-	C	-	-
	I #10	F	C, D	C	D	C, D	-	-
	I #11	F	C	C	-	C	-	(D)
	I #12	F	C	C	-	C	-	-
	I #13	M	C	C	-	C	-	-
	I #15	M	C, D	C	-	C, D	-	-
	I #17	M	C, D	C, D	D, (D)	C, D	-	-
	I #19	M	C, D	C	D	C, D	(D)	-
CONTROL	C #2	F	D	D	D	Not offered	-	-
	C #4	F	D	-	D	Not offered	-	(D)
	C #5	F	C	C	-	Not offered	-	-
	C #7	F	C	C	-	Not offered		-
	C #14	M	C	C	-	Not offered	(D)	-
	C #16	M	C, D	C, D	-	Not offered	(D)	-
	C#18	M	C, D	C, D	-	Not offered	-	-

*M* male, *F* female, *ARR* at-risk relative, *C* close relative, *D* distant relative.

^a^Presence of eligible ARR according to study documentation in the RCT. C refers to presence of one or more close relative(s) (first-degree ARRs) and D refers to presence of one or more distant relative(s) (others than first-degree ARRs). The number of close and distant ARRs was documented by the genetic healthcare professional involved in post-test counselling.

^b^Strategy used when approaching relatives. C refers to strategy used when approaching one or more close relative(s), D refers to strategy used when approaching one or more distant relative(s) (others than first-degree ARRs), (D) refers to strategy in approach to distant relatives that the patient perceived to be at risk, but the distant relative(s) were not listed as ARRs by the genetic healthcare professional.

#### 1.1 Just doing it myself

In this most common approach to risk disclosure, the patients saw it as their duty to personally inform both close and distant relatives they believed were at risk. All patients described that they informed all their close relatives themselves. Thus, even when feeling burdened by telling others about their potential risk, it was seen as something that had to be done. When disclosing to close relatives, most patients did it by telephone and soon after receiving their own counselling. Prior discussions on cancer in the family were seen as a facilitator that paved the way for talking with relatives about difficult things.

#### 1.2 Seeking assistance from family members

This subtheme reflects that some patients made shared decisions with family members on how to approach disclosure or had family members who supported them by disclosing risk information to specific relatives.

#### 1.3 Utilising the offer of direct letters

All patients in the intervention group approved of sending letters from HCP to all the ARRs for whom they had contact details available. In addition to the direct letters, they also personally communicated to all close relatives.

Three patients described that they did not inform some of their distant relatives about their possible risks - but they approved the HCP sending letters to these relatives. Hence, to these distant ARRs, the direct letter was the only means of risk disclosure from the patient. Lost contact, feelings of unease for breaking the (bad) news and not being equipped for the reactions or responding to follow-up questions were rationales for delegating the disclosure to the HCP.

#### 1.4 Vaguely relying on others to inform

This approach covers situations of passive non-disclosure, where the patient had ‘handed over’ the task of disclosing to someone else without making sure that it would be done, or the patient assumed that ARRs were already informed. When comparing interview data with the RCT-data, we saw that this passive non-disclosure was seen among patients who had misunderstood the risk for certain distant ARRs.

#### 1.5 Postponing or deciding not to

This subtheme encompassed quotes from three patients who had not (yet) conveyed information to distant ARRs that they considered were at risk. The reason for non-disclosure was lost contact, family conflict and lack of energy. Two patients had identified additional distant ARRs after cascade testing of close relatives. These distant ARRs were not listed in the RCT-data. One patient understood that an uncle and a cousin were at risk but had no contact and no plans to approach them. Another patient described that she knew testing was relevant for certain distant relatives, but due to family conflicts, she had not informed them. Both these patients expressed general ideas that in cases of lost communication in families, a healthcare-mediated disclosure option would be useful.

The third patient belonged to a family with familial breast cancer and described that she had not informed three distant relatives about their risk. The delay of information was associated with feelings of guilt but did not seem to be definitive non-disclosure, as she said that the interview had served as a reminder and (during the interview) she researched contact details for the uninformed distant relatives and now planned to send them information. According to RCT-data, these relatives were not listed as they were actually not at risk.

### Theme 2. Limiting one’s responsibility after disclosure

This theme covers the different ways of transferring perceived responsibility to the informed relatives. While many patients believed they had fulfilled their responsibility after disclosing, others continued to assist their relatives in locating testing resources or provide additional information before ‘being done’.

#### 2.1 Being done when they know

This was the most common subtheme, reflecting that if the patients had made the information available to their relatives in some form, they felt that it was now up to the relatives to decide if they wanted to act on the information or not. The view of disclosure as the end of their mission was also seen in relation to their children.

#### 2.2 They have to get counselling/testing

Some patients extended their responsibility and described being engaged in providing additional support and helped ARRs getting tested. Some older patients described a shared view within the family, where the older generation jointly tried to make sure that their adult children got tested.

#### 2.3 Providing more information

The patients’ responsibility towards ARRs could expand to prolonged engagement in managing the risk information and its consequences for the ARR, including re-contacting the HCP to address follow-up questions posed by their relatives or even arranging a meeting with the counsellor and the relatives.

### Theme 3. Being unclear about what, to whom, and by whom

Although most patients expressed being satisfied with the information initially provided, three areas of misconceptions were identified in the interaction between the cancer genetics clinic and the patients: 3.1) how to interpret the test results; 3.2) relevance for relatives and 3.3) unclear roles in risk disclosure.

#### 3.1 Misinterpretation of test results

The interviews revealed that the patients did not always know how to interpret the results of the genetic assessment. A few patients misunderstood the concept of familial risk for breast cancer or CRC, and the negative test results were interpreted as no increased risk at all, even if surveillance was offered to ARRs. There were also misconceptions that germline genetic variants could be acquired and then passed on or that inherited variants could skip generations.

#### 3.2 Which relatives are at risk?

Some quotations reflected uncertainties about whom the genetic results were relevant for, with both overestimating and underestimating the number of relatives at risk. This misunderstanding could result in telling everyone in the family or telling only the closest relatives and leaving more distant relatives without information. In general, the patients discussed the relevance of risk information to their children and siblings but expressed uncertainty regarding its relevance to more distant relatives.

#### 3.3 Who will inform the relatives?

Some interviews revealed an unclear mandate for risk disclosure between healthcare and the family. A few patients believed that their relatives would automatically be informed about the genetic assessment if they had provided clinical information to the familial assessment (i.e., given consent to confirm their cancer diagnosis). Others believed ‘someone else’ had disclosed to the ARRs or assumed that relatives already knew.

### The patients’ experiences of participating in DIRECT

Patients recalled details of the cancer genetic counselling, but their participation in the RCT seemed to play a minor role in their overall experience. When probing for study-related experiences, patients in the intervention group described accepting the offer of direct letters and none explicitly expressed integrity-related discomfort. Neither did they describe any negative reactions from relatives receiving direct letters directly from HCP. Some patients in the control group said that assistance in informing relatives would have been appreciated, but no one explicitly expressed disappointment about their treatment allocation. Misconceptions were seen about the RCT. One patient in the control group described that the HCP had offered her to directly inform her relatives if she wanted to. According to study documentation of the RCT, she did not receive that offer. Another patient allocated to the intervention group presumed that he alone was responsible for informing his ARRs, even though he had provided contact details and approved direct letters to be sent to his ARRs. Another patient believed that the intervention was standard care and that the study assessed the withdrawal of healthcare-assisted disclosure.

## Discussion

This qualitative study explored how patients perceived and carried out risk disclosure within a pragmatic RCT offering direct letters as an intervention. Consistent with a substantial body of previous research, we discovered that the patients limited their responsibility for disclosure in different ways depending on their preferences and intended audience. Novel to this study was the exploration of communication patterns in the context of participating in an RCT that offers direct letters as an alternative means for risk disclosure.

Even if all patients in the intervention group accepted the offer of having direct letters sent to their ARRs, the family-mediated disclosure to close relatives was not abandoned. In contrast, when disclosing to distant ARRs, we identified five distinct approaches ranging from ensuring that information reached everyone to delaying or choosing not to disclose. Most patients disclosed to distant relatives by personal communication and/or by delegating the task to a family member or the HCP (by accepting direct letters to be sent). These approaches were sometimes used in parallel, or sequence. Even if stating that the risk information was important to the relatives, most patients did not follow up on whether their relatives acted on the information. A few patients, however, had multiple conversations with relatives. This is in line with previous studies illustrating that disclosure is a process rather than a single act, where follow-up on behaviour also can occur [14–16]. The parallel use of direct letters and personal communication seen in our data is consistent with prior studies of patients’ attitudes reporting that patients value support when communicating with relatives and see a direct approach as a complement rather than a replacement of family communication [17, 18].

Not knowing what to say was mentioned as a real or potential barrier to disclosure - and a reason for choosing a direct letter over personal communication. We also found examples of passive non-disclosure [6], where patients assumed the ARR was already informed, or delayed disclosure because of lost contact or family conflict. Identifying communication strategies and recognising individual and interpersonal barriers and facilitators for family-mediated disclosure [19] is a crucial part of post-test genetic counselling. Finding factors motivating communication [20] and acknowledging the benefits of sharing genetic information with family members [21] could potentially affect a patient’s intention to communicate risk. Continuous engagement and provider follow-up may facilitate cascade testing procedures [22]. However, previous interventions aimed at supporting family-mediated disclosure have shown limited efficacy [23]. Ballard et al suggest that the lack of theory and insufficient involvement of the target population in intervention development are weaknesses that could contribute to the limited effect of the interventions developed so far. Mendes [24], Daly [20] and others instead emphasise the possible benefits of challenging the traditional individual genetic counselling with a family-centred approach, stressing that it may overcome barriers such as poor communication patterns within the family.

### The role of direct letters in the disclosure process

We found that all patients informed their close relatives themselves *and* approved direct letters to be sent to them. Even if information reaches the relatives, the original message may be misinterpreted in the first phase [25], and a direct letter may clarify the message or serve as a reminder. Previous research has shown that relatives who receive information from the patient alone recall significantly less accurate information compared to those receiving information directly from HCP [26].

Interventions with direct contact in a research setting has shown promising results on the uptake of counselling and testing compared to family-mediated risk disclosure [7], but the effect has not yet been proven in a clinical setting. New clinical guidelines enforcing family-mediated disclosure with follow-up, and the additional offer of direct contact into clinical practice, did not lead to an increase in the uptake of testing among ARRs in a hereditary cancer clinic [8]. Furthermore, an RCT evaluating a tailored approach where probands were offered direct contact with ARRs of cardiogenetic conditions showed no effect on uptake of testing [27]. Of note, 87% of the ARRs in that study were first-degree relatives. In this qualitative study, family-mediated risk communication was maintained to close ARRs, while some patients opted for direct letters rather than personal communication with their distant relatives. Hence, direct letters may have a more prominent role when approaching distant relatives but be redundant for close relatives. We believe that if uptake is not stratified according to degree of relation, a potential effect in certain target groups may be diluted or misinterpreted.

### Misunderstandings and different routines

All patients in this study received at least one pre- and one post-test counselling at a specialised cancer genetics clinic according to local routine. Most patients had also been provided with a family letter. Although the patients described the content of their counselling as understandable, some misunderstood both the implications of the test results and for whom this information was relevant. Our findings, along with previous reports on misconceptions [19, 28–30], strengthen the imperative that even though a sound interpretation may require genetic literacy that most people do not possess [31], patients should be provided with sufficient information to enable understanding of the implications of their genetic test results. This remains crucial, especially as genetic counselling has been described as too technical and provider-driven [32].

Cascade testing implies stepwise family communication “further out” in the family-tree. Even though being a small sample of only 17 patients, data from this study suggests a lack of uniformity in clinical routines regarding follow-up on family communication and risk disclosure. Two patients in the intervention group described active non-disclosure to correctly identified ARRs eligible for predictive testing. These ARRs were not listed as ARRs in the RCT-data. Thus, the patients did not receive the offer of HCP sending these ARRs direct letters. Clearly, if the counselling lacks identification of eligible ARRs, any direct disclosure approach will have limited effect. It is also possible that the omission of these relatives from the list of eligible ARRs (according to the HCPs), led to an unclear situation for the patients, and reduced the patients’ motivation of sharing information with them. To achieve a sustainable high standard for risk disclosure, evidence-based national guidelines on risk disclosure practices need to be developed and endorsed. To effectively implement guidelines, engagement with HCPs and research on provider barriers are necessary.

### Experiences and ethical concerns of DIRECT

Patients in the intervention group seemed to consider the offer of sending direct letters to be an integrated part of their post-test counselling rather than something that stood out. This could be related to a general expectation that healthcare will offer support in the risk-disclosing procedure, a finding seen in both our data and previous research [17, 33, 34]. We also discovered misinterpretations regarding treatment allocation (randomisation) among some of the participants and it is possible that poor understanding of the study components also contributed to unclear boundaries between what was seen as normal clinical practice and the RCT. The observations of study related misconceptions are concerning - but not surprising. A meta-analysis of the informed consent process in clinical trials shows that only about 75% of participants understand the nature of the study and the understanding of treatment allocation was even less [35]. Still, it strengthened the imperative for improving the information to consecutive potential DIRECT participants.

### Strengths, limitations and transferability of the data

We hypothesised that the patients’ experience of risk disclosure could be affected just by enrolment in the RCT, and therefore, we invited patients from both the intervention and control groups. Data collection was continued until we considered that no new information was added to the themes and subthemes. However, our findings should be seen within the context of several limitations. The sample consisted of 17 individuals, and out of those, ten individuals had been offered the intervention. The frequencies of subthemes should be interpreted with caution, considering this small sample size and that data was sourced from interactive semi-structured interviews. We do believe that the patients interviewed in this study were more prone to disclose information to their ARRs since the patient information clearly described the research procedure, and those with concerns about the direct disclosure procedure possibly declined participation in the RCT. Accordingly, another limitation is that we did not have any participants who had not shared information with any ARR. Another limitation is that the patients were interviewed about 12 months after their post-test counselling, and it is possible that their perceptions changed over time or that recall bias was introduced. Finally, we acknowledge that this study is conducted within a Swedish setting - with tax-funded healthcare and high-cost protection for patient fees- and believe that patients’ perceptions and practices of risk disclosure may differ in settings involving other healthcare systems or cultures.

### Conclusions and future research

This qualitative study revealed that the patients considered the offer of direct letters to ARR as part of standard post-test genetic counselling. The letters did not replace the process of family-mediated disclosure to close relatives. While all patients accepted the offer as a complementary route of disclosure, some patients used direct letters as the only means of communication with distant relatives.

The patients had different approaches to risk disclosure to relatives as well as different ways of limiting their responsibility after the disclosure. A lack of awareness of patients’ strategies makes it challenging to anticipate which relatives will be contacted.

Well-powered randomised quantitative studies are needed to further explore whether letters or other means of direct contact from healthcare to ARR are effective, efficient, and acceptable. We hope that our clinical trial will contribute to such knowledge. Nevertheless, besides direct letters through certified mail, other means for improving cascade testing rates should be evaluated. Health system-led risk disclosure [36], web-based applications facilitating disclosure [37], and access to genetic counselling [38] are interventions currently under evaluation. Ultimately, we need to identify a future model that balances the safety and the rights of the patient while ensuring that relatives at risk receive the relevant information.

### Supplementary information

Supplemental material

## Data Availability

The original interviews are not publicly available since that would violate the privacy of our research participants. However, the code list and working material related to the study are available from the corresponding author upon reasonable request.
