# Supplementary material for: Patients’ perceptions and practices of informing relatives: a qualitative study within a randomised trial on healthcare-assisted risk disclosure
Source: Eur J Hum Genet. 2024 Feb 2;32(4):448–55. doi: 10.1038/s41431-024-01544-8 (PMC10999412; doi:10.1038/s41431-024-01544-8)
Supplement: Supplementary file 1 — Supplemental material [file 41431_2024_1544_MOESM1_ESM.docx]

**Supplementary information on methodology**

[Data collection 2](#_Toc145696880)

[Phase 1 (February to June 2021) 2](#_Toc145696881)

[Phase 2 (October to December 2021) 3](#_Toc145696882)

[Detailed descriptions of the analytical procedure 4](#_Toc145696883)

[Ontological and epistemological assumptions 6](#_Toc145696884)

[Axiological assumptions and researchers’ backgrounds 6](#_Toc145696885)

[Interview guide version 1.0 8](#_Toc145696886)

[Interview guide version 2.0 9](#_Toc145696887)

## Data collection

### Phase 1 (February to June 2021)

This qualitative study recruited patients who participated in a clinical trial (The DIRECT Study, ClinicalTrials.gov Identifier: NCT04197856). At the time of inclusion in the trial, participants were informed that they might be invited to an interview later. One year after the start of the trial, in February 2021, we invited the first 22 consecutively recruited patients for interviews. Six of them were unreachable, but all others agreed to participate, resulting in 14 conducted interviews. Two interviews were excluded from the analysis; one because of failed recording and one because our internal pilot evaluation determined that the informant did not meet the recruitment criteria of being offered a test for hereditary breast, ovarian or colorectal cancer (she was a proband with familial risk of breast cancer, but the (negative) genetic testing with a breast cancer panel was performed on another family member).

Interviews following a semi-structured interview guide were performed by author Jenny von Salomé (JvS) (Interview guide version 1.0). To be open to the participants’ experiences and to minimize preconceptions, the interviewer was provided with only the participants’ contact details. Hence, the interviewer was blinded to both clinical information (e.g., patient’s medical history, family diagnosis, and number of ARR) and research-related information (e.g., allocation to intervention/control). All interviews were conducted over the telephone, recorded, and transcribed verbatim by an administrator experienced in transcribing interviews. The transcripts were checked in detail against the recordings by the first author Charlotta Nääs (CN).

In the preliminary analysis of the data (conducted in September 2021 by CN, JvS and AR, see details below), we identified misconceptions among the respondents regarding, for example, which relatives were at risk. This pattern was of great interest because of the following reasons: 1) we aimed to explore how the participants performed and perceived risk disclosure, and misconceptions may affect disclosure and 2) misconceptions may be both a safety issue and a methodological consideration in the clinical trial. We also interpreted that the respondents seemed to limit their responsibility for disclosure in different ways. To further elaborate on the misconceptions and respondents’ limits of responsibility, we extended our data collection by conducting additional interviews.

### Phase 2 (October to December 2021)

Before the second phase of data collection, the interview guide was further revised (Interview guide 2.0). To allow for clearer follow-up questions, the interviewer was no longer blinded to clinical and health-related information of the patients, including the number of identified relatives at risk. For the second data collection, we deliberately invited informants to ensure maximum variation in gender and family diagnoses. In this phase, CN conducted interviews with five men who were identified as carriers of PV in *BRCA1*, *BRCA2*,  *PALB2* or the *MMR*-genes between October and December 2021. Reflective notes were used to capture initial thoughts and ideas from the interviews. The interviews were transcribed by CN. After reading the five transcripts, we concluded that the added interviews had given more insight into how the patients limited their responsibility for risk disclosure in different ways. No additional misconceptions about study participation were seen, but misunderstandings about the implications of genetic testing persisted in the data. At this point, we considered the data collection rich enough to reflect a variation in the experiences of participants enrolled in the DIRECT Study. There was no previous relationship between the participants and the interviewer. The interviews lasted 28 to 55 minutes. We did not repeat any interviews and the transcripts were not returned to the participants.

## Detailed descriptions of the analytical procedure

The transcripts of all included interviews from the first phase (n=12) were read individually by all co-authors and preliminary ideas and reflections were noted individually. Next, all three authors met for two full days in September 2021 and cooperatively developed initial codes for the first two interviews. During this coding process, we developed initial semantic codes (close to the original articulation of the text). However, we also identified hidden meanings and added latent initial codes. When coding the rest of the material, the main coder (CN) labelled the text with both semantic and latent codes. All coding was performed in Open Code 4.03. After coding the first 12 interviews, all codes were exported to a Word document. To get a sense of the representativeness of the different codes, codes related to each interviewed patient were given a unique colour. At the second meeting between the authors (October 2021), we organised the codes according to their association with different topics and later grouped the codes with shared meanings into different initial themes. At this point, we reflected that data related to the overall experience of the DIRECT study was not very rich. In fact, it seemed to have a minor impact on their overall experience and was rather seen as part of the care offered to them. Nevertheless, we wanted to present this part of the data and chose to organise the data related to the study separately.

Analysis continued during 2022, both individually by CN and during joint author meetings between March and September 2022. As the analysis continued, we went back and forth between the original data to develop subthemes and themes. Using two Excel sheets (one for each phase of data collection), we re-organised the codes and reconstructed some of the themes. We continued to keep codes related to the RCT experience separate from the other codes.

In a parallel process, we examined how the different topics and initially constructed themes were represented in the data. For this, we developed an overview of the data in Excel with our interpretation of each interview. The following topics were addressed: 1) general impression of the interview; 2) how the patient had come into contact with the cancer genetic unit; 3) the patient´s general attitudes towards risk disclosure; 4) if and how the patient had disclosed to relatives and if so, to whom; 5) if misconceptions seemed to be present; 6) whether the patient described having used healthcare-mediated disclosure; 7) whether the patients described that they had received help to disclose from another party; and 8) how the patients seemed to limit their responsibility. In the overview, we also added quantitative data from the study documentation in the RCT (age, gender, family diagnosis, study group allocation, and number of ARR).

Throughout the process of writing the article, codes related to each theme were kept in the draft, allowing us to keep close to the original codes while writing. As the analysis evolved, we drafted (several) conceptual maps of how the patients perceived and performed risk disclosure. However, this mapping did not add understanding to the underlying meaning of the themes. Therefore, we decided to present two themes related to how the patients perceived and practised risk disclosure, one theme reflecting patients’ misunderstandings, and one separate section about our interpretation of their overall experience of the DIRECT Study. For the text related to the study, we applied descriptive analysis. The overall approach and the main analysis follow the step of reflexive thematic analysis. At submission to European Journal of Human Genetics, reviewers suggested revising and expanding the theme on how the patients performed risk disclosure (Theme 1. Sharing risk with unpredictable outcomes), especially in relation to the intervention offered. It was also suggested to explore if the frequency of sub-themes differed between study arms or other characteristics. In August and September 2023, we therefore went back to the previous overview of the interviews, re-read the original data and codes for each interview and thereafter quantified frequencies of subthemes in Theme 1. We also revised the description of Theme 1 and omitted the less novel data on motivation for sharing information from the theme. Risk disclosure behaviour was a topic in the original interview guide and thus, the subthemes of Theme 1 were discussed in detail in all interviews. In the contrary, Theme 2 and 3 developed during analysis, and therefore, their subthemes were not suitable for frequency estimates.

## Ontological and epistemological assumptions

In data interpretation, we focused on the participants’ perceptions and actions as described by themselves. We assumed that the participants’ experiences could be reflected in their language and generated semantic codes close to the text. However, when we interpreted an underlying meaning of the language, we also constructed latent codes (with a higher level of interpretation). We used inductive data inquiry as reflected in our decision to continue data collection after the first preliminary analysis.

## Axiological assumptions and researchers’ backgrounds

We want to acknowledge that even though we intended to distance ourselves from our own preconceptions and personal experiences, our preconceptions and experiences may have affected how we interpret the data or how we present the results. The main coder (CN) was a medical student at the time of data collection and coding. We purposively decided that she should avoid reading the literature of similar studies until coding was finalised. Therefore, we believe that CN was suitable for this purpose as she had not been engaged in genetic counselling nor had she any previous academic background in clinical genetics or cancer genetics (apart from medical school). JvS is a PhD and has worked as a genetic counsellor in cancer genetics for more than 15 years. AR is a clinical geneticist working at the cancer genetic clinic from where most the patients were recruited; and is the PI for the DIRECT Study. She has previously written an article arguing that HCP has a role to play in risk disclosure to ARRs (Grill K, Rosén A. Healthcare professionals’ responsibility for informing relatives at risk of hereditary disease. J Med Ethics. 2021;**47:**e12). That is, AR is engaged in cancer genetics with ongoing research on risk disclosure of hereditary cancer.

For JvS and CN, this was the first time they were engaged in a qualitative research project. AR has worked with qualitative analyses (grounded theory and qualitative content analysis) in previous research projects

## Interview guide version 1.0

WAY IN

How did the cancer genetic investigation start for you?

Has your family talked about cancer risk before?

What do you think about the fact that it could be a hereditary risk?

Did you understand what that meant?

DISCLOSURE

Do you have any relatives affected by the investigation?

If you have told them . . . Can you tell HOW and WHEN you informed your relatives?

how did it feel?

what information have you chosen to include?

how did they react?

how did you handle questions you received from them?

RESPONSIBILITY

What are your thoughts about relatives being affected?

Did it feel like you had a responsibility to inform them?

Have you contacted more distant relatives?

How have you experienced the role/task of informing about genetic risk in the family?

Do you feel “done” with informing?

DIG IN TO: MORAL/LEGAL

MOTIVATION

Was it important to you to tell your relatives and why? How did you decide what you wanted to tell them?

HYPOTHETICAL QUESTIONS:

What do you generally think about informing people about their hereditary risk?

If a person is notified that there is an increased risk in the family but does not pass the information on, what do you think about it?

If the person distinctly refuses to pass on information to relatives who have an actual risk, what do you think about it?

If you found out that a relative knew about a significant risk of disease but did not tell you, how would you feel?

Do you think there is a responsibility to inform about hereditary risk in the family?

Who has that responsibility?

If you had the choice to let the healthcare system inform your relatives, do you think it would be an interesting option?

TIP

Did you get any support in informing?

What kind of support would you like to have to make it easier for you to inform the family?

In what format would such support be offered?

WHAT would such information say?

In which SITUATION do you think it would be reasonable for healthcare to take on the responsibility of informing?

INTERVENTION

How did you experience that offer?

Are you missing something in the care within the framework of the heredity investigation?

Is there anything else you would like to address/other thoughts you have about this?

QUANTITATIVE QUESTIONS:

Which of your relatives did you perceive this to be important to? How many of the affected relatives have you told?

Were you told who ELSE might be affected by the risk? Who were you expected to tell?

Quantitative question: Who have you told this to in general (i.e., not just relatives)?

## Interview guide version 2.0

INTRO/BACKGROUND

Why was the heredity investigation started?

What did they say? Do you have a hereditary risk?

How did it feel to get the message?

Did you understand what it meant? Do you have a diagnosis?

Do you have any relatives affected by the investigation (identify which ones and how many at-risk relatives)?

Have your family talked about cancer risk before?

INFO

If you did tell, can you tell HOW you informed your relatives? WHICH ones?

Did you get any help informing them?

Intervention group only: What did the assistance include (e.g., thinking that healthcare takes care of everything and knowledge that letters were sent as registered mail)? How were relatives identified? How were the addresses found?

How did it feel to disclose the information? How did the relatives react?

Do you feel “done” with the disclosure?

RIGHTS OF RELATIVES/OWN OBLIGATIONS

Why did you tell your relatives?

Was it important for you to tell them and why?

How did you decide what you wanted to tell them?

Do you think your relatives have the right to access the information (and/or right to no information)?

Have you contacted more distant relatives?

Did it feel like you had a responsibility to inform them?

What is the boundary of your responsibility based on: perceived closeness to the relative? When did the relative receive the information? When did \they contact cancer genetics? When were you included in the control program?

Who has the ultimate responsibility that the relatives receive the information (you, another relative, or healthcare)?

RESPONSIBILITY, LAW, AND MORALITY

(First open and general, then specific)

1. What do you generally think about informing people about their hereditary risk?

2. Do you think there is a responsibility to inform about hereditary risk in the family?

3. Who has that responsibility?

4. What do you think if a person is notified that there is an increased risk in the family but does not pass on the information?

If the person distinctly refuses?

If you found out that a relative knew about a hereditary risk but did not tell you, how would you feel?

For whose sake do you do a heredity investigation?

Does the person who has undergone an investigation have any obligations?

SUGGESTIONS OF IMPROVEMENT

Was there anything that made it/did not make it difficult to disclose? (e.g., own disease, family size, and help from others)?

Control group: If you had the choice to let the healthcare system inform the family, do you think it would be an interesting option?

Intervention group: How did you perceive the offer that healthcare could contact relatives?

What kind of support would you like to have to make it easier for you to inform the family (format and content)?

Is there any situation where healthcare should inform the relatives?

ENDING

Are you missing something within the framework of the heredity investigation?

Is there anything else you would like to address, or do you have other thoughts you have about this?

Do not forget to have received answers to:

Which of your relatives did you perceive this to be important to?

How many of the affected relatives have you told?
